# TGFβ signaling pathway is altered by HLA-B27 expression, resulting in pathogenic consequences relevant for spondyloarthritis

**DOI:** 10.1186/s13075-024-03370-1

**Published:** 2024-07-15

**Authors:** Marc Lauraine, Maxence de Taffin de Tilques, Dganit Melamed-Kadosh, Bilade Cherqaoui, Vincent Rincheval, Erwan Prevost, Aurore Rincheval-Arnold, Eneida Cela, Arie Admon, Isabelle Guénal, Luiza M. Araujo, Maxime Breban

**Affiliations:** 1 Infection & Inflammation, UMR 1173, Inserm, UVSQ, Université Paris Saclay, 2 avenue de la Source de la Bièvre, Montigny-le-Bretonneux, 78180 France; 2 Laboratoire d’Excellence Inflamex, Université Paris Cité, Paris, France; 3 Université Paris-Saclay, UVSQ, LGBC, Versailles, 78000 France; 4 Department of Biology, Technion-Israel Institute of Technology, Haifa, 32000 Israel; 5 Service de pédiatre, Hôpital Ambroise Paré, AP-HP, Boulogne-Billancourt, France; 6 Rheumatology, allergology and clinical immunology, University of Rome “Tor Vergata”,, Rome, Italy; 7 Service de rhumatologie, Hôpital Ambroise Paré, AP-HP, 9 avenue Charles de Gaulle, Boulogne, 92100 France

**Keywords:** Spondyloarthritis, BMP/TGFβ pathway, HLA-B27, CD4 + T cells, Peptidome, Rat, *Drosophila*

## Abstract

**Background:**

Association of HLA-B27 with spondyloarthritis (SpA) has been known for 50 years, but still remains unexplained. We recently showed that HLA-B27 expressed in wing imaginal disc from HLA-B27/human-β2 microglobulin (hβ2m) transgenic *Drosophila* deregulated bone morphogenetic protein (BMP) pathway by interacting physically with type I BMP receptor (BMPR1) Saxophone (Sax), leading to crossveinless phenotype.

**Methods:**

Genetic interaction was studied between activin/transforming growth factor β (TGFβ) pathway and HLA-B27/hβ2m in transgenic *Drosophila* wings. The HLA-B27-bound peptidome was characterized in wing imaginal discs. In mesenteric lymph node (mLN) T cells from HLA-B27/hβ2m rat (B27 rat), physical interaction between HLA-B27 and activin receptor-like kinase-2 (ALK2), ALK3 and ALK5 BMPR1s, phosphorylation of small mothers against decapentaplegic (SMADs) and proteins of the non-canonical BMP/TGFβ pathways induced by its ligands, and the transcript level of target genes of the TGFβ pathway, were evaluated.

**Results:**

In HLA-B27/hβ2m transgenic *Drosophila*, inappropriate signalling through the activin/TGFβ pathway, involving Baboon (Babo), the type I activin/TGFβ receptor, contributed to the crossveinless phenotype, in addition to deregulated BMP pathway. We identified peptides bound to HLA-B27 with the canonical binding motif in HLA-B27/hβ2m transgenic *Drosophila* wing imaginal disc. We demonstrated specific physical interaction, between HLA-B27/hβ2m and mammalian orthologs of Sax and Babo, i.e. ALK2 and ALK5 (i.e. TGFβ receptor I), in the mLN cells from B27 rat. The magnitude of phosphorylation of SMAD2/3 in response to TGFβ1 was increased in T cells from B27 rats, showing evidence for deregulated TGFβ pathway. Accordingly, expression of several target genes of the pathway was increased in T cells from B27 rats, in basal conditions and/or after TGFβ exposure, including *Foxp3*, *Rorc*, *Runx1* and *Maf*. Interestingly, *Tgfb1* expression was reduced in naive T cells from B27 rats, even premorbid, an observation consistent with a pro-inflammatory pattern.

**Conclusions:**

This study shows that HLA-B27 alters the TGFβ pathways in *Drosophila* and B27 rat. Given the importance of this pathway in CD4 + T cells differentiation and regulation, its disturbance could contribute to the abnormal expansion of pro-inflammatory T helper 17 cells and altered regulatory T cell phenotype observed in B27 rats.

**Supplementary Information:**

The online version contains supplementary material available at 10.1186/s13075-024-03370-1.

## Introduction

Spondyloarthritis (Spa) is a chronic inflammatory rheumatism that can affect axial and/or peripheral skeleton. It typically combines sacroiliitis with spondylitis, which can progress into bone fusion. It may also comprise peripheral arthritis and enthesitis, dactylitis, and extra-articular features, including uveitis, psoriasis, and inflammatory bowel disease (IBD). The HLA-B27 allele of the Class-I major histocompatibility complex (MHC-I) is the genetic factor most strongly associated with SpA, being present in 70–90% of the patients [1].

This striking association was first described 50 years ago but its underlying mechanisms remain incompletely understood [2]. Several lines of rats transgenic for HLA-B27 and human β2-microglobulin (hβ2m) (B27 rat) were developed to investigate the implication of HLA-B27 in the pathogenesis of SpA. The B27 rats develop spontaneously multisystem inflammatory disorder that resembles human SpA by combining arthritis with IBD and psoriasis and is specific for HLA-B27 as HLA-B7/hβ2m transgenic rats remain healthy. Among other advances, this model highlighted the critical role of myeloid derived cells expressing HLA-B27 and CD4 + T cells in disease development [3]. In particular, an uncontrolled expansion of pro-inflammatory T helper 17 (Th17) cells and an altered regulatory T cell (Treg) phenotype, depending on deregulated antigen-presenting cells function were evidenced in this model [4–6]. In contrast, CD8 + T cells appeared dispensable, arguing against the “arthritogenic” peptide hypothesis, which implicated the canonical antigen-presentation function of the MHC-I molecule.

Recently, we produced a transgenic *Drosophila* expressing HLA-B27 to identify HLA-B27 non-canonical properties that could explain its pathogenicity [3]. In this new model, we reported the unexpected interference of HLA-B27 with the bone morphogenetic proteins (BMP)/transforming growth factor beta (TGFβ) pathway. Indeed, when HLA-B27 (but not HLA-B7) was expressed in combination with hβ2m in the *Drosophila* wing imaginal discs, it was shown to physically interact as well-folded conformer (recognized by w6/32 antibody (Ab)) with the type I BMP receptor (BMPR1) Saxophone (Sax) and to antagonize its inhibitory function thereby enhancing BMP signaling and leading to a loss of crossveins. Likewise, physical interaction was specifically observed between well-folded conformers of HLA-B27 (recognized by ME1 and w6/32 Abs) and activin receptor-like kinase-2 (ALK2, the Sax mammalian ortholog) in B-lymphoblastoid cell lines (B-LCL) from SpA patients. Moreover, stimulation of T cells from HLA-B27 + SpA patients with both TGFβ and activin-A led to an exacerbated response [7].

The BMP/TGFβ pathway is one of the most pleiotropic signaling pathways. It is involved in embryonic development as well as in adult organisms in various phenomena such as homeostasis, cell migration, and cell proliferation. It is notably implicated in ossification and immune response, two complementary facets of SpA pathology [8]. TGFβ1 exerts major effects on the differentiation of naïve CD4 + T cell (Tn), by promoting their differentiation into Treg and Th17 cells [9–11]. The right balance between these two subsets of CD4 + T cells appears critical to prevent several autoimmune/inflammatory diseases including SpA [12]. Given the excessive Th17 expansion and altered Treg phenotype that were observed in B27 rats and the critical role of activin/TGFβ signaling in Treg/Th17 differentiation [4, 5], here, we investigated whether activin/TGFβ pathway was altered in HLA-B27 transgenic *Drosophila* and in CD4 + T cells from B27 rats.

## Materials and methods

### Drosophila crossing and wings preparation

Fly strains were grown in standard corn/agar medium. Crosses were performed at 25 °C. The different fly stocks employed in this study are listed in Supplementary Table 1. Wings were dissected and placed in a glycerol/ethanol drop on a glass slide and imaged at 4X magnification using an Olympus SZX16 stereomicroscope and an Olympus Infinity3 camera. Veins length and wing area were measured with FIJI pixel length and area functions (https://imagej.nih.gov/ij/).

### Drosophila wing imaginal discs collection

Drosophila wing imaginal discs were collected as described in [13]. Briefly, this protocol enables the recovery of many wing imaginal discs by grinding a large quantity of *Drosophila* larvae followed by organs separation using filtration and a density gradient. To ensure sufficient HLA-B27 recovery after affinity purification for high-quality bound-peptides analysis, we determined that approximately 2 × 10^8^ cells expressing HLA-B27 were necessary. As imaginal wing disc contains 50,000 cells and 60% of imaginal wing disc cells express HLA-B27 [7], 7,000 nub > HLA-B27 and 4,000 wild-type *Drosophila* wing imaginal discs were collected. Collected wing imaginal discs were frozen at -80 °C and sent to the Technion-Israel Institute of Technology for HLA-B27-bound peptidome identification.

### Affinity purification of the HLA molecules and analysis of the bound peptides

Total proteins were extracted from wing imaginal discs and the HLA molecules were immunoaffinity-purified using the w6/32 mAb, which recognizes HLA-A, B, and C well-folded heavy chain- covalently linked to AminoLink-agarose resin (Thermo Fisher Scientific, Rockford, IL) as described previously [14]. The HLA molecules with their bound peptides were eluted from the beads with 1% trifluoroacetic acid (TFA), which also induces dissociation of the HLA-β2m peptide complexes. The released peptides were separated from the HLA heavy subunit, the β2m, and from other bound proteins using disposable reversed-phase MicroTip C18 columns (Harvard Apparatus, Holliston, MA) and eluted with 30% acetonitrile and 0.1% TFA, as described previously [15] while the HLA heavy chain, β2m, and other bound proteins were recovered with 30% acetonitrile and 0,1% TFA. The peptides were partially resolved by a capillary high-performance liquid chromatography (HPLC) on pulled capillaries of 0.075-mm inner diameter and about 20 cm long [16] packed with C18 reversed-phase 3.5-µm beads (Reprosil-C18-Aqua, Dr. Maisch GmbH, Ammerbuch-Entringen, Germany). Chromatography was performed with the UltiMate 3000 RSLC nano-capillary UHPLC system (Thermo Fisher Scientific), which was coupled by electrospray to tandem mass spectrometry (MS) on Q-Exactive-Plus (Thermo Fisher Scientific), using the same parameters as in Bourdetsky et al., 2014 [17]. The HLA peptides were eluted with a linear gradient over 2 h from 5 to 28% acetonitrile with 0.1% formic acid at a flow rate of 0.15 µl/min. Data were acquired using a data-dependent ‘top 10’ method, fragmenting the peptides by higher energy collisional dissociation (HCD). The full scan MS spectra were acquired at a resolution of 70,000 at 200 m/z with a target value of 3 × 10^6^ ions. Ions were accumulated to AGC target value of 10^5^ with a maximum injection time of 100 millisec. No fragmentation was performed for peptides with unassigned precursor ion charge states or charge states of four and above. The peptide match option was set to Preferred. Normalized collision energy was set to 25%, and MS/MS resolution was set to 17,500 at 200 m/z. Fragmented masses were dynamically excluded from further selection for fragmentation for 20 s.

The protein fraction eluted from the affinity column was eluted from the same reversed-phase column with 80% acetonitrile. The eluted protein fractions were dried by vacuum centrifugation, dissolved 8 M urea (Sigma-Aldrich), 400 mM ammonium bicarbonate (Sigma-Aldrich) and 10 mM dithiothreitol (Sigma-Aldrich). Half of the sample was reduced at 60 °C for 30 min. Carbamidomethylation was performed in the dark for 30 min by 40 mM iodoacetamide (IAA, Sigma-Aldrich). Next, three volumes of HPLC water were added, followed by 0.2 µg trypsin (Promega, Madison, Wisconsin, USA) and an overnight incubation at 37℃. Another aliquot of 0.2 µg trypsin was added and incubated for 3 h at 37 °C. The digested samples were acidified to a final concentration of 0.1% TFA and desalted on C18 StageTips prior to MS analysis. The tryptic peptides from the 80% acetonitrile fraction described above were analyzed in liquid chromatography (LC)-MS/MS using Q Exactive HF mass spectrometer (Thermo) fitted with a capillary HPLC easy nLC 1200 (Thermo-Fisher Scientific). The peptides were loaded in solvent A (0.1% formic acid in water) on a homemade capillary column (30 cm, 75-micron ID) packed with Reprosil C18-Aqua (Dr. Maisch GmbH, Germany). The peptides mixture was resolved with a 5 to 28% linear gradient of solvent B (80% acetonitrile with 0.1% formic acid) for 120 min followed by a gradient of 15 min of 28 to 95% and 15 min at 95% acetonitrile with 0.1% formic acid in water at flow rates of 0.15 µl/min. Mass spectrometry was performed in a positive mode (m/z 300–1800, resolution 60,000 for MS1 and 15,000 for MS2) using repetitively full MS scan followed by HCD fragmentation at 27 normalized collision energy of the 10 most dominant ions with two and above charges selected from the first MS scan. The AGC settings were 3 × 10^6^ for the full MS and 1 × 10^5^ for the MS/MS scans. The intensity threshold for triggering MS/MS analysis was 1 × 10^4^. A dynamic exclusion list was enabled with an exclusion duration of 20 s.

### Peptidome data analysis

Peptides were identified and quantified using the MaxQuant software tool [18] version 1.6.17.0 with the Andromeda [19] search engine using the *Drosophila melanogaster* section of the UniProt/Swiss-Prot database (release Jan 29, 2021, containing 22,114 entries, 13,821 genes). HLA peptides were identified in the database assuming no specific enzyme proteolysis. Methionine oxidation and N-acetylation were accepted as variable modifications. The peptide precursors and fragment mass tolerances were set at 6 and 20 ppm, respectively. The minimal peptide length was set to eight amino acid residues. The false discovery rate (FDR) was set, separately, for 0.05 for HLA peptides. The ‘match between runs’ subroutine was used in the analysis. MaxQuant quantifies the relative signal intensities of the peptides using their LC-MS peak volumes. Graphical and statistical analyses of the results were performed with Perseus [20]. Assignment of HLA scores to the different identified peptides was done by NetMHC, which ranks the peptides according to their fitness to one of the HLA allomorphs, with peptides ranking below 2% relative to 400,000 different peptides in the NetMHC database considered as intermediate affinity ligands of the particular HLA [21, 22].

The raw MS files of the trypsin-digested protein (80% acetonitrile) fractions were also analyzed by MaxQuant version 1.6.3.4 and searched with the Andromeda search engine using the HLA-B27 and human β2m sequences added to the Drosophila melanogaster section of the UniProt/Swiss-Prot database. The search settings were trypsin-specific, with FDR set to 0.01 and decoy mode Revert. Methionine oxidation and N-acetylation were accepted as variable modifications, while carbamidomethylation of cysteines was accepted as a fixed modification. Intensity-based absolute quantification (iBAQ) was used to quantify the levels of purified HLA-peptide complexes.

The MS peptidomics and proteomics data have been deposited to the ProteomeXchange Consortium [23] (http://proteomecentral.proteomexchange.org) via the PRIDE partner repository with the dataset identifier PXD047119.

### Rats

The SpA-prone B27 rats of the 33-3 line bearing 55 copies of HLA-B*2705 and 28 copies of hβ2m and the healthy B7 rats of the 120-4 line bearing 52 copies of HLA-B*0702 and 26 copies of hβ2m, all on a Fisher (F344) background, were bred under conventional conditions [24]. Age-matched nontransgenic (NTG) littermates were used as controls. Rats were used at 2 distinct ages: (i) 3–5 wk, asymptomatic, so-called premorbid; (ii) 1,5–12 mo, adults presenting disease symptoms. Study procedures were approved by the Institutional Animal Experimentation Ethical Committee from the Faculty of Health Simone Veil (APAFIS-8910).

### Rat cells preparation

Single-cell suspensions were prepared from mesenteric lymph nodes (mLN), stained with appropriate Abs and analyzed by flow cytometry with BD LSR Fortessa. In some experiments, total T cell population was gated using anti-CD3 antibody (Ab) (Fig. S1C). In other experiments, appropriate combinations of Abs were used to identify and/or sort Tn (CD4 + CD25-CD62L^high^) and effector CD4 + T cells (Teff; CD4 + CD25-CD62L-) (Fig. S1D).

### Proximity ligation assay

Proximity ligation assay (PLA) was performed using Duolink In Situ PLA reagents from Merck (Red detection reagent duo 92,008/Probe Anti-Rabbit PLUS 92,002/Probe Anti-Mouse MINUS 92,004) following the manufacturer’s protocol. For each experiment, 3 × 10^5^ total mLN cells were used. Primary Abs w6.32 (Abcam) and CD45RC (Bio-rad) are produced in mice, anti- ALK2 (Sigma-Aldrich), anti- ALK3 (Invitrogen) and anti- ALK5 (Sigma-Aldrich) are produced in rabbit. They were all used at 1:100 except for ALK5 that was used at 1:150. Whole volume of approximately 10 random fields of view were acquired with 63x oil immersion objective of Leica SP8 confocal microscope using the DAPI (blue nuclei) and TexasRed (Red PLA signal) filters. PLA signal was manually counted in each individual cell and for each Z field using LasX software.

#### RT-qPCR

Tn and Teff from NTG and B27 rats were collected in TRIzol RNA Isolation Reagents (ThermoFisher), then RNA was extracted using classic chloroform/isopropyl alcohol extraction protocol. cDNA was synthesized by reverse transcription using RevertAid First Strand cDNA Synthesis Kit (ThermoFisher) with polyA primers, then real time quantitative polymerase chain reaction (RT-qPCR) was performed using SsoAdvanced™ Universal SYBR^®^ Green Supermix (BioRad) and CFX384 (Touch Real-Time PCR Detection System). Duplicates were run for each sample; *Gapdh* was used as endogenous reference gene for mRNA abundance normalization. Primer sequences are provided in Supplementary Table 3.

#### Cell stimulation

Single-cell suspensions (10^6^ cells) from mLN were stimulated with recombinant human (rh) TGFβ1 (5ng/ml) (Miltenyi Biotec) or PBS for 1 h at 37 °C/5% CO2. In experiments using sorted-cells, the stimulation was performed on single-cell suspension (5.10^5^ or 10^6^ cells) after 1 h of cell resting. When sorted, cells were left to rest at 37 °C/5% CO2 for 1 h.

#### Th17 cell differentiation assay

For Th17 differentiation experiment, flow cytometry-sorted Tn (CD4^+^CD25^-^ CD62L^+^) were cultured (10^5^ cells/well) in the presence of coated-anti-CD3 (2 µg/ml), soluble anti-CD28 (1 µg/ml), neutralizing anti-IFN-γ and anti-IL-4 Abs (each at 10 µg/mL), and various combinations of rhTGFβ1 (2 ng/ml), recombinant rat (rr) IL-1β, rrIL-6 and/or rrIL-23 (each at 20 ng/mL). All Abs were purchased from ThermoFisher and rr cytokines from R&D Systems. At day 3, the medium was replaced by fresh medium supplemented with rrIL-2 (20 ng/mL). At day 6, supernatants were harvested for analysis of IL-17 production by ELISA.

#### SMAD2/3, p38, AKT, ERK, mTOR, NFκB phosphorylation assessment by flow cytometry

Single-cell suspension or sorted CD4 + T cell subsets isolated from NTG or B27 rats mLN, were stimulated or not with rhTGFβ1 for 1 h as described previously. After stimulation, the cells were fixed for 20 min in Fix Buffer I (BD Bioscience) and then permeabilized for 20 min in Perm Buffer III (BD Bioscience) at -20 °C. Intracellular staining with specific Abs was performed for 1 h in Perm Wash 1x according to the manufacturer’s instructions (BD Bioscience). Unstimulated and unsorted, cells were fixed immediately after isolation. Results were expressed as staining index (SI) calculated as the ratio between samples and fluorescence-minus-one staining control.

### Statistical analysis

Data are expressed as the mean ± SEM. Kruskal-Wallis test, followed by Dunn’s multiple comparisons tests comparing each condition to its control was used to analyse *Drosophila* experiments. Chi-2 test was used to compare the second position residue between peptides specific for B27 drosophila and those non-specific. Paired t-tests or Fischer’s LSD tests were used to analyse flow-cytometry experiments. Unpaired t-tests were used for qPCR experiments. Analyse of variance (ANOVA) followed by unpaired t-test after Bonferroni correction was used for PLA experiment. Results were considered significant when p value was ≤ 0.05.

## Results

### HLA-B27 interacts genetically with the activin/TGFβ pathway in *Drosophila*

**Fig. 1:**
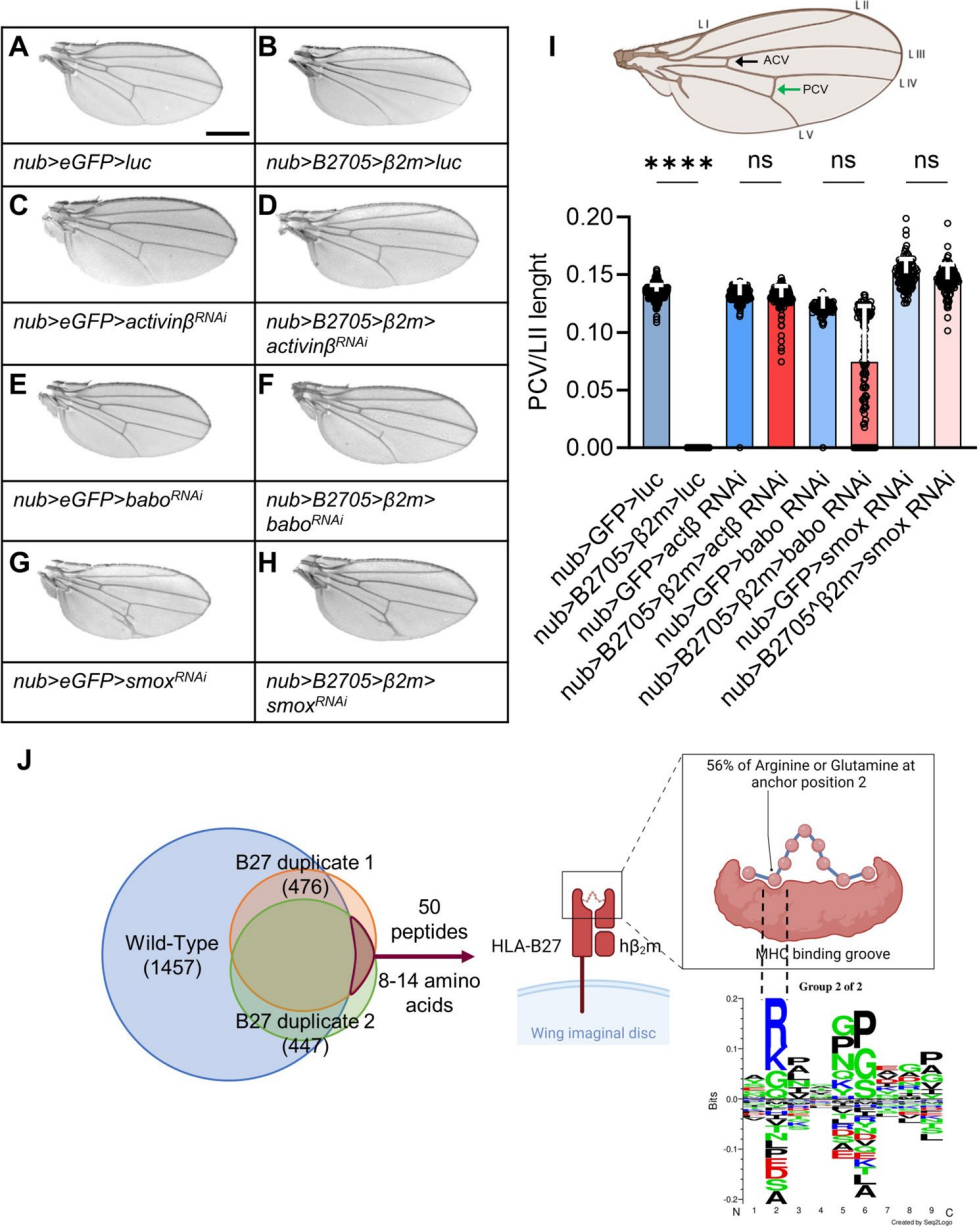
HLA-B*27:05/hβ2m expressed in *Drosophila* wing genetically interacts with the activin pathway and binds specific peptidome. (A-H) Nubbin-GAL4 (nub-GAL4) was used to drive transgenes expression in the pouch of imaginal wing disc. Scale bar: 500 μm. eGFP and luciferase were used as controls and have no effect on wing formation. Adult fly wings expressing genotypes: (**A**) nub > enhanced Green fluorescent protein (eGFP) > luciferase (luc) (nub-GAL4/+; UAS-eGFP/UAS-luc), (**B**) nub > HLA-B27 > hβ2m > luc (nub-GAL4/+; UAS-HLA-B*27:05, UAS-hβ2m/UAS-luc), (**C**) nub > eGFP > activinβ (actβ) RNA interference (RNAi) (nub-GAL4/+; UAS-eGFP/UAS-actβRNAi), (**D**) nub > HLA-B27 > hβ2m > actβRNAi (nub-GAL4/+; UAS-HLA-B*27:05, UAS-hβ2m/UAS-actβRNAi), (**E**) nub > eGFP > baboon (babo)RNAi (nub-GAL4/+; UAS-eGFP/UAS-baboβRNAi), (**F**) nub > HLA-B27 > hβ2m > baboRNAi (nub-GAL4/+; UAS-HLA-B*27:05, UAS-hβ2m/UAS-baboRNAi), (**G**) nub > eGFP > smoxRNAi (nub-GAL4/+; UAS-eGFP/UAS-smoxβRNAi), (**H**) nub > HLA-B27 > hβ2m > smoxRNAi (nub-GAL4/+; UAS-HLA-B*27:05, UAS-hβ2m/UAS-smoxRNAi), (**I**) *Drosophila* wing: longitudinal veins (L I to L V), ACV Anterior CrossVein (black arrow), PCV Posterior CrossVein (green arrow), Scatter plot with bar analysis of PCV/LII length ratio, *n* > 80, Kruskal-Wallis followed by Dunn’s multiple comparisons test showing only a difference between the control and B*27:05/hβ2m conditions (****: *p* < 0.0001). Vertical error bars represent SD. (**J**) HLA-B*27:05 from duplicate samples of *Drosophila* wing imaginal discs was retained on immunoaffinity column, using w6/32 Ab. Analysis of the peptides eluted from the column, revealed 50 peptides considered as specifically bound to HLA-B27, as present in both B27 *Drosophila* duplicates and absent from wild-type *Drosophila* wing imaginal disc (left panel). The logo motif (created with GibbsCluster; right panel) shows a high proportion of peptides carrying an Arginine, R or Glutamine, Q in second position that is characteristic of HLA-B27 peptidome (28/50, 56%). A significantly lower proportion of peptides shared between wild-type imaginal discs and B27 duplicates exhibited similar characteristic (28/721, 3.8%; *p* < 0.0001) (figures created with BioRender)

We previously reported that HLA-B27, when expressed in combination with hβ2m in *Drosophila* wing imaginal disc, physically interacted with BMPR1 saxophone (Sax) leading to an increased BMP signaling and a loss of crossveins [7]. Given the possible interplay between BMP and activin/TGFβ pathways that signal *via* Thickveins (Tkv)/ Sax and baboon (Babo) BMPR1s, respectively, we further investigated the involvement of the latter pathway in the loss of crossveins induced by HLA-B27/hβ2m expression. Down regulation of the ligand of the pathway, *activin*, or its BMPR1, *Babo*, or its signal transducer *Smox* using RNA interference (RNAi) do not affect wing formation in wild-type *Drosophila* (Fig. 1A,C,E,G). However, in B27/hβ2m *Drosophila*, it leads to a full or partial rescue of the crossveins, confirming a genetic interaction between HLA-B27 and the activin pathway in addition to the BMP pathway (Fig. 1B,D,F,H,I). Altogether, this indicates that in the presence of HLA-B27, inappropriate activin/TGFβ signaling participated to the loss of crossveins.

### Well-folded conformers of HLA-B27/hβ2m loaded with canonical peptides are expressed in transgenic *Drosophila* wing imaginal disc

In our previous work, we showed that HLA-B27 folded and localized to the cell membrane in *Drosophila* wing imaginal disc. Its recognition by ME1 Ab suggested that the HLA-B27 peptide-binding groove was not empty [7]. To further address this question, we collected wing imaginal discs from *Drosophila* expressing HLA-B27/hβ2m. Wild-type wing imaginal discs were used as control. Immunoaffinity purification of HLA-B27 molecule was performed with w6/32 antibody, followed by large scale quantitative MS to identify HLA-B27-specific peptide ligands. In this assay, both HLA-B27 and hβ2m molecules were purified from the HLA-B27/hβ2m *Drosophila* wing disc and absent from the wild-type condition (according to the proteome analysis of the 80% acetonitrile fraction, showing clear identification and quantification of both proteins). Furthermore, peptidome analysis identified 50 peptides specific for HLA-B27 (Fig. 1J, left panel) with 56% presenting an arginine (50%) or a glutamine (6%) at the anchor position 2, a hallmark of HLA-B27 peptide-binding motif (Fig. 1J, right panel). These peptides are longer on average than those usually purified from mammalian cells with a majority spanning 10 to 11 amino acid residues (61%) and < 1% having the canonical 9 amino acids in length. Altogether these findings indicate that HLA-B27 associates with both hβ2m and specific peptides in *Drosophila* despite the lack of the peptide loading complex and ERAP aminopeptidase, which might explain the longer than usual length of those peptides, as previously shown [25]. The *Drosophila* proteins and functions corresponding to these peptides as well as their binding affinity predicted by NetMHC 4.0 software are shown in Table 1. According to its rank, QRAMNPVMSI, a peptide derived from Fat-body protein 1, isoform D was predicted as strong binder to HLA-B27, whereas 4 other peptides were predicted as weak binders. Noteworthy, a comparison of HLA-B27-bound *Drosophila* peptides with previously reported HLA-B27 peptidome from human or B27 rat cells revealed only one rat peptide overlapping with one of the *Drosophila* peptides (TEAPLNPKAN), both derived from actin. However, in the rat, it was not specific for the HLA-B27-bound peptidome [25, 26].

**Table 1:** HLA-B27-specific peptides in *Drosophila* with their corresponding protein and implication in biological process

Peptide	Second amino acid	Binding affinity (nM) NetMHC 4.0	Rank NetMHC 4.0*	Protein	Biological process
APGSGAPG	P	42,924	95	Trailer hitch, isoform E	Cytoskeleton
EEQEAEVDEN	E	43350.6	99	Tubulin beta-1 chain	
GRPLVTMSTY	R	209.4	0.8	Uncharacterized protein, isoform E	
LEELQQENCR	E	40662.7	85	Augmin complex subunit dgt3	
TEAPLNPKAN**	E	42344.2	95	Actin-5 C	
NRLMYSVIGATEY	R	4131	5	Z band alternatively spliced PDZ-motif protein 66, isoform I	
VQYDPRNSETY	Q	22,065	22		
VRYQQQQQQQQQY	R	4863.8	5.5	Z band alternatively spliced PDZ-motif protein 52, isoform R	
AQGDFNEFIEK	Q	26901.4	31	Neuropeptide-like 2	Immunity
ARNDPQDDAE	R	27782.9	33	GH02216p	
ARNDPQDDAEVIK	R	13252.4	12		
GRITTNQDR	R	2115.8	3.5	Fat-body protein 1, isoform D	
QRAMNPVMSI	R	106.7	0.5		
SRMEYSFSNGVVTQ	R	1483.1	3	Sidestep III, isoform O	
SRLTYGSNA	R	1596.4	3	Fondue, isoform A	
SRLTYGSNAASTF	R	1088.6	2.5		
SSRLTYGSNAASTF	S	3689.4	5		
ARAEINFEGPSP	R	9277.4	8.5	BcDNA.GH11973	DNA/RNA
ARAEINFEGPSPA	R	4606.2	5.5		
ARPYQPDGYQY	R	13923.4	12	RE50839p	
NGEFGNELPQRQ	G	39358.9	80	Vig2, isoform B	
STITSREIQ	T	34397.8	55	Histone H2B	
SVNQQSKTQTVSN	V	34054.2	55	Protein lingerer, isoform L	
AAKQTGPIVIS	A	33461.8	55	40 S ribosomal protein S19a (Ribosomal protein S19a, isoform C)	Translation
AGDSKANPPKGAA	G	36829.9	70		
EEIKKEVSS	E	37291.4	70		
GQIANGYTPV	Q	4211.7	5	Elongation factor 1-alpha	
NPNQQSERP	P	37283.8	70	Eukaryotic translation initiation factor 4H1, isoform A	
VRIGQPIMSV	R	983.7	2.5	60 S ribosomal protein L10	
DGSVGPVGP	G	41344.1	90	Nicotinic acetylcholine receptor alpha7, isoform A	Ion transport
DGSVGPVGPAGP	G	42269.1	95		
HTNNVQEPK	T	14523.8	13	Calsyntenin-1	
TVSGVNGP	V	39174.1	80	V-type proton ATPase subunit B (Vacuolar proton pump subunit B)	
YSKTKSAAPNFDE	S	28671.3	35	GEO09626p1	
EGVEGIEHQE	G	43,278	99	Protein GDAP2 homolog	Stress response
IEQAGKDATEN	E	42,286	95	Cytochrome b5	
TEDASHMEEVD	E	42945.4	95	Heat shock protein 83	
ERNQNGEDVVK	R	18,746	17	FI19426p1	Membrane receptor
QRVGITAEDL	R	1000.7	2.5	Msr-110, isoform D	
YRNAGLYNGNT	R	8464.9	8	GM18993p	
YRNAGLYNGNTL	R	743.8	1.8		
YRNAGLYNGNTLVD	R	566.5	1.5		
GRSNISFATSP	R	7799.6	7.5	Nucleoporin NUP53	Nuclear membrane
APSGGPAG	P	44078.1	99	HECT-type E3 ubiquitin transferase	Ubiquitylation
GRGNIGQTNY	R	970.3	2.5	3-oxoacyl-[acyl-carrier-protein] reductase	Fatty acid metabolism
SRAPGGAGTGGM	R	7840.5	7.5	Protein transport protein Sect. 61 subunit beta	Autophagy/ Intracellular protein transport
GPGAGPGQRP	P	40685.1	85	FI16123p1	Unknown
MRVPPYYLLM	R	554	1.5	Uncharacterized protein, isoform A	
MRVPPYYLLMQ	R	3821.6	5		
YRIIESNEVPK	R	874.3	2	Uncharacterized protein	

Flybase website was used to determine the corresponding proteins and biological process (flybase.org)

*Rank NetMHC 4.0: Rank threshold for strong binding peptides: 0.500 / Rank threshold for weak binding peptides: 2.000

**This peptide was shared with the HLA-B27-bound peptidome isolated from B27-rats spleen but was not specific for HLA-B27 [25]

### HLA-B27 interacts with ALK2 and ALK5 in B27 rat

**Fig. 2:**
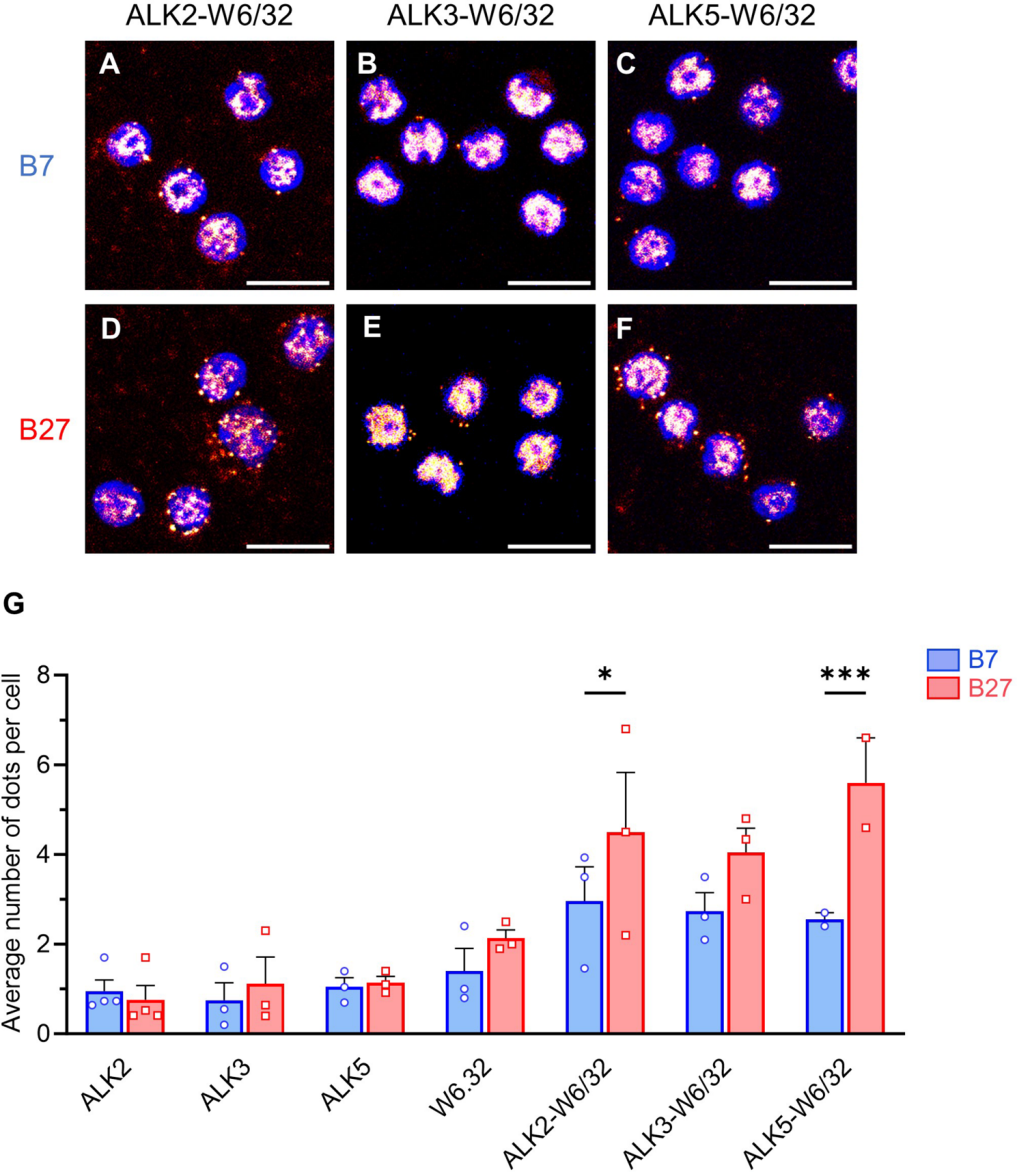
HLA-B27 interacts with ALK2 and ALK5 in mLN lymphocytes from B27 rats. PLA was performed on lymphocytes isolated from (**A-C**) B7 or (**D-F**) B27 rats mLN, using w6.32 pan MHC-I Ab and anti-ALK2, -ALK3 or -ALK5. Images show representative field for each condition. Cells were stained for nuclei (Blue, DAPI) and PLA signal dots (RedHot, PLA kit probes). Scale bar: 10 μm. Images were treated with Fiji software. An unspecific PLA signal was homogeneously observed in all conditions. (**I**) Average number of PLA staining dots/cell in 2 to 4 independent experiments, showing a greater interaction between ALK2 or ALK5 and HLA-B27 than HLA-B7, whereas interaction was similar for ALK3 with both alleles. The background signal using single Abs was similar to the signal observed by combining anti-ALK Abs with anti-CD45RC (Supplementary Fig. 1). In conditions combining two Abs, approximately 300 to 400 cells per experiment were manually counted, whereas 150 to 250 cells per experiment were manually counted in single Ab conditions. Two-way ANOVA (factors: Ab and rat strain) followed by Bonferroni’s multiple-comparisons test was performed (*: *p* < 0.05 ***: *p* < 0.001). Vertical error bars show SEM

**Fig. 3:**
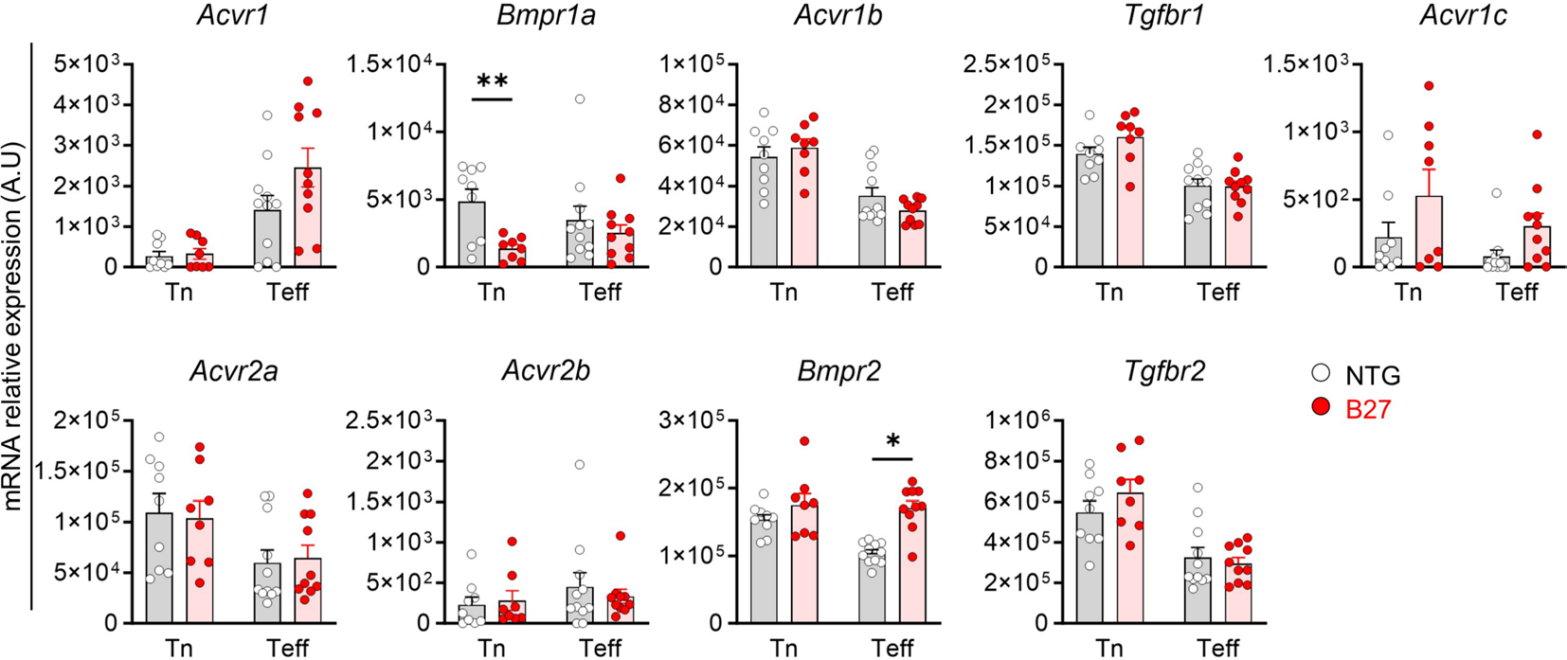
BMP/TGFβ pathway receptors expression in Tn and Teff from B27 and NTG rats. The mRNA expression of TGFβ and activin receptor genes was assessed by RT-qPCR in ex-vivo sorted Tn and Teff from NTG (grey bar – white dots) and B27 (red bar – red dots) adult rat mLNs. Scattered dot plot with bars represents the mean of mRNA relative expression in arbitrary units (A.U) out of 8 to 11 experiments. Differences between NTG and B27 expression levels for each receptor were tested by unpaired t-tests with Welch’s correction (**: *p* < 0.01). Error bars show SEM. On the first row *Acvr1*, *Bmpr1a*,* Acvr1b*,* Tgfbr1 and Acvr1c* genes coding for ALK2, ALK3, ALK4, ALK5 and ALK7 BMPR1s respectively. On the second row *Acvr2a*,* Acvr2b*,* Bmpr2 and Tgfbr2* represent type 2 BMP receptor genes

The mammalian orthologs of the three *Drosophila* BMPR1s, Sax, Tkv and Babo are ALK2, ALK3 and ALK5, respectively. They are primarily involved in BMP (ALK2 and ALK3) and Activin/TGFβ (ALK5, also known as TGFβR1) signaling, respectively. We have previously shown a specific physical interaction between HLA-B27 and ALK2 in B-LCLs derived from SpA patients. Here, we wished to investigate the physical interaction between HLA-B27 and ALK2, ALK3 and ALK5 in B27 rat mLN cells, using PLA and HLA-B7 transgenic rats as control. This assay revealed a significantly greater interaction of ALK2 and ALK5 with HLA-B27 than HLA-B7 (Fig. 2A,C,D,F,G). In contrast, the level of interaction of ALK3 with both HLA-B molecules was similar (Fig. 2B,E,G). The PLA signal between ALKs and irrelevant CD45RC protein was comparable to background (Supplementary Fig. 1A,B,C). These PLA results could not be explained by variations in the levels of expression of HLA-B27 and HLA-B7 which were similar in both lines of rats [27]. Moreover, the levels of expression of the different BMPRs were similar between B27 and control rats, except for a decreased expression of *Bmpr1a* that codes for ALK3 in Tn and an increase of *Bmpr2* in Teff from B27 rat (Fig. 3).

### TGFβ induces heightened phosphorylation of SMAD2/3 in T cells from B27 rat

**Fig. 4:**
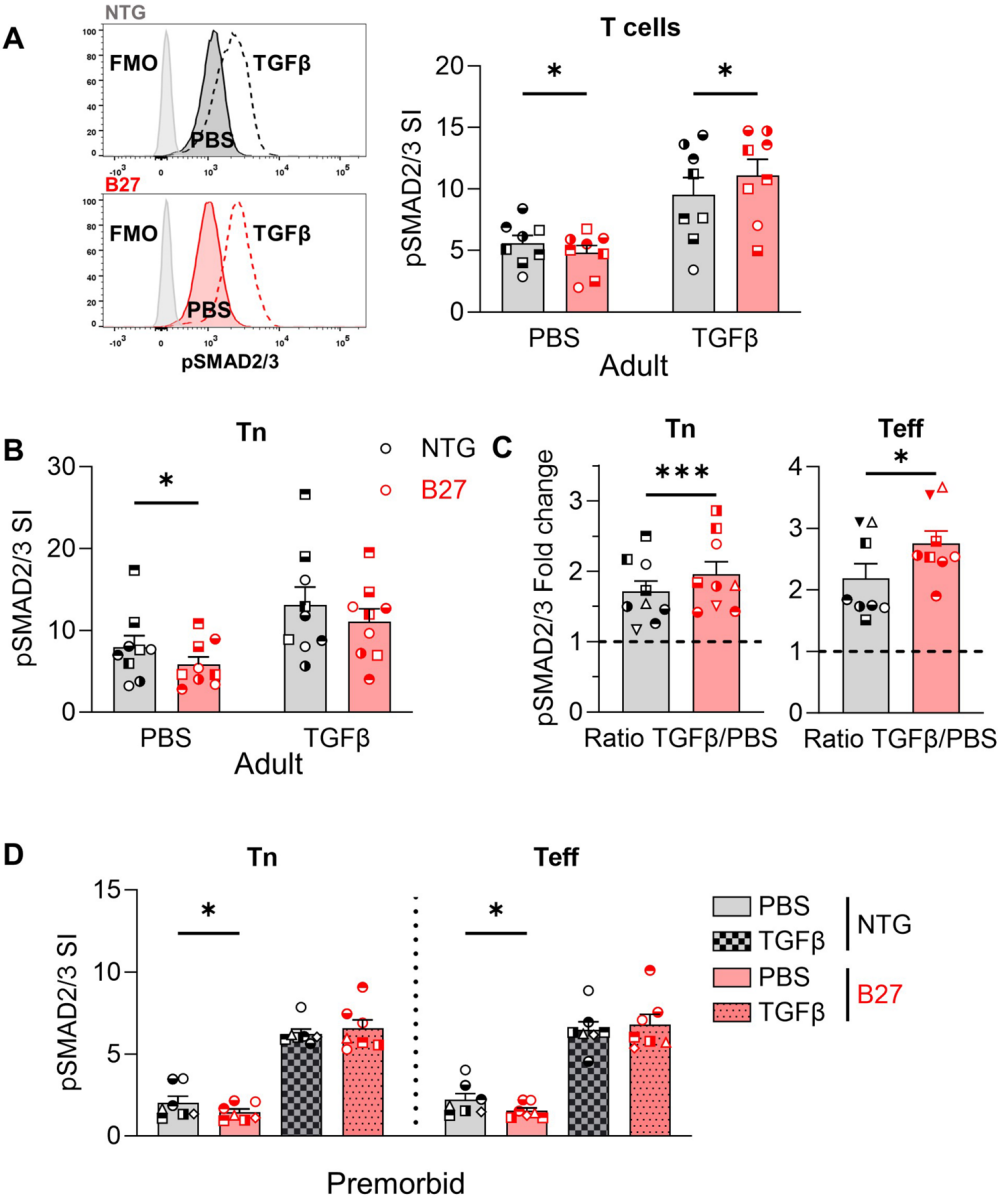
Basal pSMAD2/3 is reduced in B27 rat T cells but increases more after TGFβ1 stimuation. (**A**) Left: Representative histograms of pSMAD2/3 expression in mLN T cells from NTG and B27 rats after 1 h exposure to medium alone (PBS) or TGFβ1 at 37 °C. Right: Intracellular pSMAD2/3 was quantified in mLN T cells from B27 and NTG adult rats after exposure to TGFβ1 or medium alone. Scatter dot plots with bars representing the mean of the SI of 8 experiments. Similar symbols indicate paired conditions. Vertical error bars show the SEM. Paired t-test *: *p* < 0.05. (**B**, **C**) Intracellular pSMAD2/3 was quantified in Tn (**B**, **C**) or sorted Teff (**C**) from B27 and NTG adult rats after exposure to medium (PBS) or TGFβ1 in 8 to 9 independent experiments. Similar symbols indicate paired conditions. (**B**) Bars height shows the SI mean values. Vertical error bars show the SEM. Paired t-test *: *p* < 0.05. (**C**) Mean of pSMAD2/3-fold change after exposure to TGFβ expressed as the ratio of pSMAD2/3 SI after TGFβ1 stimulation over the SI without exposure from (**B**) (left panel) and from sorted Teff (right panel). Paired t-test *: *p* < 0.05; ***: *p* < 0.001, (**D**) Intracellular pSMAD2/3 was quantified after TGFβ1 stimulation or no stimulation by gating on Tn or Teff from premorbid NTG and B27 rats. Scatter dot plots with bar show the pSMAD2/3 SI. Similar symbols indicate paired conditions. Bars height show the SI mean values (*n* = 7–9 independent experiments). Vertical error bars show the SEM. Paired t-test *: *p* < 0.05

To test whether the BMP/TGFβ pathway was affected by expression of HLA-B27 in adult rat, we stimulated bulk mLN cells or sorted CD4 + T cell subsets with BMPs (2, 4 or 6), activin A or TGFβ1. BMPs failed to induce the phosphorylation of SMAD1/5/8 in rat T cells after different doses and timing of exposure (Supplementary Fig. 2A). Likewise, activin A had a very little or no effect on rat T cells (Supplementary Fig. 2B). In contrast, TGFβ1 increased SMAD2/3 phosphorylation in all mLN T cell subsets. The phosphorylation of SMAD2/3 in CD3 + T cells was significantly lower in unstimulated mLN cells from adult B27 rats and higher after TGFβ1 treatment (Fig. 4A; Supplementary Fig. 2C,F), an observation similar to what we already reported in peripheral blood T cells from SpA patients [7]. In sorted Tn, the level of SMAD2/3 phosphorylation was also lower in unstimulated cells from adult B27 rats than in NTG rat, but after TGFβ1 treatment, phosphorylation reached comparable levels between NTG and B27 rats (Fig. 4B; Supplementary Fig. 2D). In B27 Teff, SMAD2/3 phosphorylation after stimulation with TGFβ1 almost reached significant difference compared to NTG Teff (*p* = 0.06) (Supplementary Fig. 2D). Thus, the pSMAD2/3-fold change (i.e. TGFβ/PBS ratio) was higher in both Tn and Teff from B27 rats (Fig. 4C and Supplementary Fig. 2F).

We next examined the effect of TGFβ1 on SMAD2/3 phosphorylation in mLN T cell subsets from 3 weeks-old premorbid rats to eliminate the impact of chronic inflammation on this response. Again, in medium control conditions, a lower level of SMAD2/3 phosphorylation was observed in Tn but also in Teff from B27 rats compared to their NTG littermates (Fig. 4D, Supplementary Fig. 2E). The level of pSMAD2/3 achieved after TGFβ1 exposure in Tn and Teff was similar between B27 and NTG rat but, as for adult rats, the fold-change was greater in B27 rat (Fig. 4D and Supplementary Fig. 2G).

Besides canonical SMAD2/3 phosphorylation, TGFβ may signal through non-canonical pathways. To address the effect of TGFβ on these pathways, we tested the phosphorylation of p38, AKT, mTOR, ERK1/2 and NF-κB after TGFβ1 treatment, in gated or sorted mLN T cell subsets from adult and premorbid B27 rats, in comparison with NTG control rats. Whatever the time of exposure, TGFβ1 failed to induce phosphorylation of any of these proteins (Supplementary Fig. 3A). However, basal phosphorylation of some of them was significantly decreased in several premorbid B27 rat T cell subsets. Thus, the basal levels of p-p38, pAKT, pmTOR, and pERK were significantly lower in Tn and/or Teff from premorbid B27 rats than in NTG littermates (Supplementary Fig. 3B). This was generally not the case in T cells from adult rats.

### Differential expression of TGFβ1 target genes in Tn from NTG and B27 rats

**Fig. 5:**
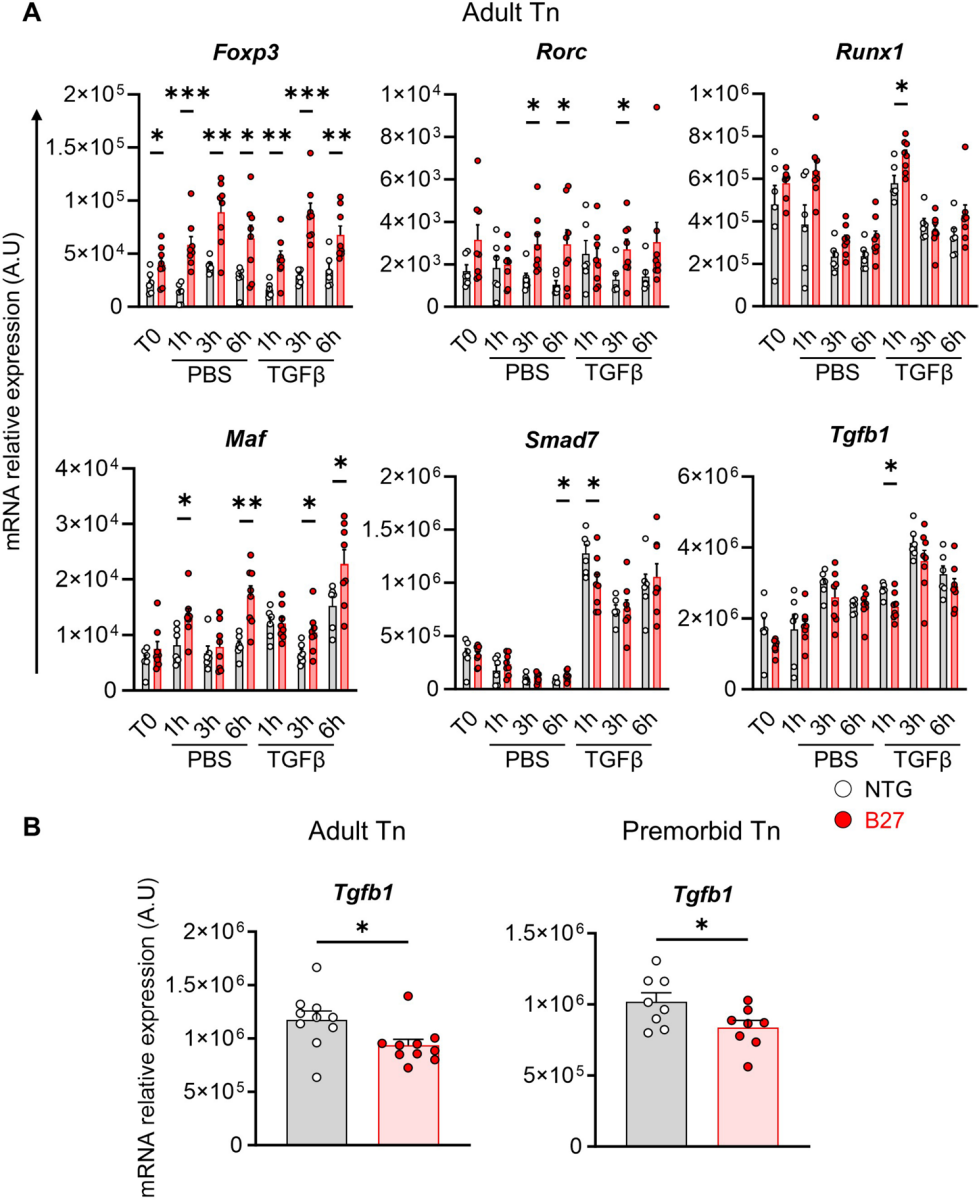
TGFβ target genes are differentially expressed between NTG and B27 rat Tn. (**A**) mRNA relative expression in mLN-sorted Tn from NTG (grey bar – white dots) or B27 (red bar – red dots) adult rats immediately after sorting (T0) or after 1 h, 3 h and 6 h of incubation, without (PBS) or with TGFβ1, assessed by RT-qPCR. Scattered dot plots with bars show the mean of the mRNA relative expression out of 6 NTG and 8 B27 experiments. (**B**) *Tgfb1* mRNA relative expression in ex-vivo sorted Tn from NTG and B27 adult (left panel) and premorbid (right panel) rats was assessed by RT-qPCR. Scattered dot plot with bars show the mean of mRNA relative expression out of 10 experiments for adult rats and 8 experiments for premorbid rats. In all graphics, differences between NTG and B27 expression level was tested by Welch’s t-tests, *: *p* < 0.05; **: *p* < 0.01; ***: *p* < 0.001

We next addressed whether higher SMAD2/3 phosphorylation amplitude observed after TGFβ1 exposure in several T cell subsets from B27 rats translated into differential target genes induction. Kinetic of target genes transcripts level was quantified in sorted Tn from adult B27 rats and NTG littermates, after exposure to TGFβ1 over 6-hrs. In Tn from adult B27 rats, expression of *Foxp3* and *Rorc*, which are characteristic TGFβ target genes, was increased in basal condition but not modified by TGFβ1 treatment (Fig. 5A). *Runx1* that codes for a transcription factor implicated in Th17 cell differentiation, in combination with RORγt and FOXP3, reached a significantly higher level of expression in B27 rat, after 1 h of TGFβ1 exposure, coincident with its peak of expression (Fig. 5A). Expression of *Maf* that is also implicated in Th17 cell differentiation was also higher in Tn from B27 than NTG rats with a noticeable peak of expression in B27 Tn after 6 h induction. In contrast, two other TGFβ target genes, *Smad7*, which codes for an inhibitor of the pathway and *Tgfb1* itself were less induced by TGFβ, in B27 than in NTG rat Tn after 1 h of TGFβ1 exposure but reached comparable levels thereafter, which indicates slightly different kinetics of stimulation (Fig. 5A). Given the importance of TGFβ itself in controlling inflammation, we investigated the *Tgfb1* expression in ex-vivo sorted Tn and revealed a lower expression in both adult and premorbid rats B27 rats compared to NTG rats (Fig. 5B).

### Tn from B27 rats produce heightened levels of IL-17 under Th17 polarizing conditions

**Fig. 6:**
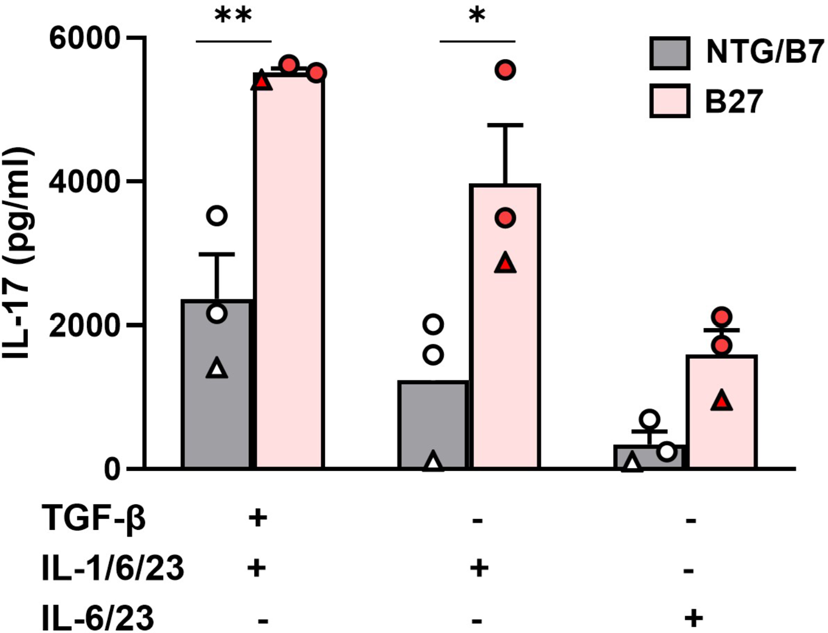
B27 rats Tn cultured under Th17 polarizing conditions produce heightened levels of IL-17. Sorted-naive T cells (CD4^+^CD25^−^CD62L^+^) from B27 or control (NTG-circle or B7-triangle) rats were cultured in Th17 differentiation conditions as indicated in figure legend. At day-6, IL-17 levels were evaluated in supernatants by ELISA. Values are mean + SEM of 3 rats/group. Paired t test * *p* < 0.05 **p < 0.005

Next, we investigated whether increased expression of *Runx1*, *Rorc and Maf* genes in sorted Tn might alter their capacity to differentiate in Th17 cells in optimal (TGFβ + IL-1 + IL-6 + IL-23) or sub-optimal (IL-1 + IL-6 + IL-23 or IL-6 + IL-23) differentiation conditions. The Th17-differentiation from B27 rats Tn, resulted in higher levels of IL-17 production in all conditions of culture as compared to differentiation from control (NTG or B7) rats Tn (Fig. 6).

## Discussion

Using a new model of HLA-B27/hβ2m transgenic *Drosophila* to explore non-canonical effects of HLA-B27 that might explain its pathogenicity, we previously reported an interaction between HLA-B27 and Sax, one of three *Drosophila* BMPR1s. Our previous study showed that HLA-B27 expressed in wing imaginal disc exerted a dominant negative effect on Sax function, limiting BMP signaling during crossveins formation, resulting in a loss of crossveins by enhanced BMP signaling [7]. Moreover, HLA-B27 conformers recognized by w6/32 and ME1 Abs were shown to interact physically with Sax in wing imaginal disc and also with its mammalian orthologue ALK2 in B-LCLs from SpA patients.

In the present follow-up study, we first evidenced that the loss of crossveins induced by expression of HLA-B27/hβ2m in *Drosophila* wings implicates a deregulated activin/TGFβ pathway. Hence, the ligand activin, its BMPR1 receptor Babo and their down-stream transcription factor Smox were all required for the crossveinless phenotype, indicating inappropriate signaling through the activin/TGFβ pathway in the presence of HLA-B27/hβ2m. This suggests a complex interplay between HLA-B27 and the BMPR1s, which are known to form different combinations of homo or heterodimers with variable outcome [28]. In addition, we evidenced that at least some of the HLA-B27/hβ2m conformers purified from the wing imaginal disc with w6/32 Ab were loaded with peptides enriched in canonical B27-binding motif. This may support the conclusion that well-folded HLA-B27 conformers are involved in the wing phenotype. However, the identified peptides did not overlap with previously characterized B27-specific peptidome from human or B27 rat cells. Thus, if the mechanism by which HLA-B27 alters wing formation in *Drosophila* is related to its role in SpA pathogenesis, this is unlikely to involve the binding of defined peptides.

We next turned to the B27 rat to examine the possible implication of BMP/TGFβ pathway in SpA pathogenesis. This murine model is characterized by an uncontrolled expansion of pathogenic Th17 cells and a deregulated phenotype of Treg [4, 5]. We have recently shown that Tn from B27 rats were prone to develop a pro-inflammatory Th17 phenotype and that this happened even before disease development [29]. One of the key mechanisms behind the biased differentiation of B27 rat Tn relied on decreased expression of interferon/Th1-related genes, including *Stat1* and *Tbx21*, that was already apparent in CD4 + single-positive thymocytes from premorbid B27 rats [29]. This could account for heightened Th17 differentiation since transcription factors from both pathways cross-regulate each other.

Thus, given the known implication of the BMP/TGFβ pathway and particularly of TGFβ in the differentiation of CD4 + T cells into Th17 cells, by inhibiting expression of the master transcription factors of Th1 differentiation, including *Tbx21* [9, 30–33] it was interesting to determine if inappropriate signaling *via* this pathway could be the molecular event linking HLA-B27 to the foregoing signature. Possible relevance of such hypothesis was first supported by PLA showing close physical interaction of ALK2 and ALK5, the respective orthologs of Sax and Babo, with HLA-B27/hβ2m at the membrane of rat lymph node cells, superior to that with HLA-B7/hβ2m, whereas interaction of ALK3 with both HLA-B alleles was comparable. However, despite the expression of their type 1 and type 2 receptors at the RNA and/or protein levels in rat T cells, neither BMPs nor activin A induced detectable phosphorylation of SMAD1/5/8 or SMAD2/3, respectively. In contrast, TGFβ induced canonical SMAD2/3 phosphorylation in rat T cells. We observed consistent decreased basal phosphorylation in B27 rat T cell subsets, whereas phosphorylation increased to a greater extent, reaching at least comparable or greater levels after TGFβ1 treatment. Overall, this is suggestive of enhanced TGFβ signaling in T cells from B27 rats.

We next examined the putative consequences of these findings on several TGFβ target genes and made several interesting observations: two key target genes, i.e. *Foxp3* and *Rorc* were increased in Tn from B27 rats without stimulation but were not further modified until 6 h following TGFβ exposure, whereas two other genes, *Runx1* and *Maf* were induced to higher levels in Tn from B27 rats with TGFβ. This is all the most interesting that the combination of those 4 gene products is critical to determine the fate of Tn, particularly regarding Th17 and Treg differentiation [34, 35]. Consistently, we observed that differentiating Th17 from B27 rats Tn cells resulted in heightened IL-17 production whatever the combination of cytokines added for polarization. The increased basal levels of the first two genes could be a consequence of in vivo exposure of Tn from B27 rats to TGFβ. This interpretation is consistent with our previous epigenomic study of Tn from B27 rats that evidenced *de novo* binding motifs for transcription factors including SMAD3 and SMAD4, that were predicted to preferentially bind to B27 rat-specific superenhancer regions [29]. Moreover, ingenuity pathway analysis of RNAseq data from adult and premorbid B27 Tn cells predicted TGFβ1 as an upregulator of genes differentially expressed as compared to NTG Tn cells ([29] and unpublished data). In turn, the decreased basal phosphorylated state of SMAD2/3 in T cells from B27 rats could be interpreted as the consequence of negative feedback following increased signaling and induction of phosphatases [8, 36]. This basal difference could potentially explain delayed induction of *Smad7* and *Tgfb1* in B27 rat T cells after exposure to TGFβ. It could possibly also explain that *Tgfb1* transcripts were decreased in ex vivo sorted Tn, which could bear direct consequences on the development of SpA, given the critical importance of T cell-produced TGFβ1 to prevent chronic inflammation [37].

The precise molecular mechanism by which HLA-B27 may impact BMP/TGFβ signaling both in *Drosophila* and mammalian cells is still unknown. However, it is noticeable that hβ2m plays a critical role in the development of abnormal phenotype in both animal models that we studied, i.e. the HLA-B27 transgenic *Drosophila* and the B27 rat [3, 7]. Interestingly, β2m has been shown to bind and signal through TGFβ receptors inducing proinflammatory pathway [38]. Thus, it is possible that the propensity of HLA-B27 molecule to interact physically with BMPR1s, including ALK5 could favor signaling by β2m through TGFβ receptor.

## Conclusions

Our study shows that HLA-B27/hβ2m expressed in *Drosophila* induces detrimental TGFβ signalling. Deregulated TGFβ signalling was also observed in CD4 + T cells from B27 rat that could account for pathogenic Th17 differentiation, a hallmark of SpA. Aberrant physical interaction between well-folded HLA-B27/hβ2m complex and BMP/TGFβ type I receptors, could account for such deregulation.

### Electronic supplementary material

Below is the link to the electronic supplementary material.

Supplementary Material 1

## Data Availability

Peptidome data are available via ProteomeXchange with identifier PXD047119.

## Abbreviations

SpA: Spondyloarthritis
hβ2m: Human β-2-microglobulin
B27 rat: Rat transgenic for HLA-B27 and hβ2m
BMP: Bone morphogenetic protein
BMPR1: Type 1 bone morphogenetic protein receptor
Sax: Saxophone
TGFβ: Transforming growth factorβ
mLN: Mesenteric lymph nodes
ALK: Activin receptor-like kinase
SMAD: Small mothers against decapentaplegic
Babo: Baboon
IBD: Inflammatory bowel disease
MHC-I: Class I major histocompatibility complex
Th17: T helper 17
Treg: Regulatory T cell
Ab: Antibody
B-LCL: B-lymphoblastoid cell line
Tn: Naive CD4 + T cell
TFA: Trifluoroacetic acid
HPLC: High-performance liquid chromatography
MS: Mass spectrometry
HCD: Higher energy collisional dissociation
LC: Liquid chromatography
FDR: False discovery rate
iBAQ: Intensity-based absolute quantification
NTG: Nontransgenic
Teff: Effector CD4 + T cell
PLA: Proximity ligation assay
mAb: Monoclonal antibody
rh: Recombinant human
rm: Recombinant mouse
rr: Recombinant rat
RT-qPCR: Real-time quantitative polymerase chain reaction
PBS: Phosphate buffer saline
SI: Stain index
ANOVA: Analysis of variance
Tkv: Thickveins
RNAi: RNA interference
UAS: Upstream activating sequence
