# Supplementary material for: TGFβ signaling pathway is altered by HLA-B27 expression, resulting in pathogenic consequences relevant for spondyloarthritis
Source: Arthritis Res Ther. 2024 Jul 15;26:131. doi: 10.1186/s13075-024-03370-1 (PMC11247877; doi:10.1186/s13075-024-03370-1)
Supplement: Supplementary file 1 — Supplementary Material 1 [file 13075_2024_3370_MOESM1_ESM.docx]

**Supplementary Fig. 1: ALK2 and ALK3 do not interact with CD45RC.** PLA was performed on lymphocytes isolated from (A) B7 or (B) B27 rats mLN, using anti-ALK2 or anti-ALK3 with anti-CD45RC irrelevant antigen. Images show representative field for each genotype in the ALK2-CD45RC condition. Results were similar in ALK3-CD45RC condition. Cells were stained for nuclei (Blue, DAPI) and PLA signal dots (RedHot, PLA kit probes). Scale bar: 10µm. Images were treated with Fiji software. (C) Average number of PLA staining dots/cell on 150 to 200 cells in one experiment, showing no difference with results using single Abs.


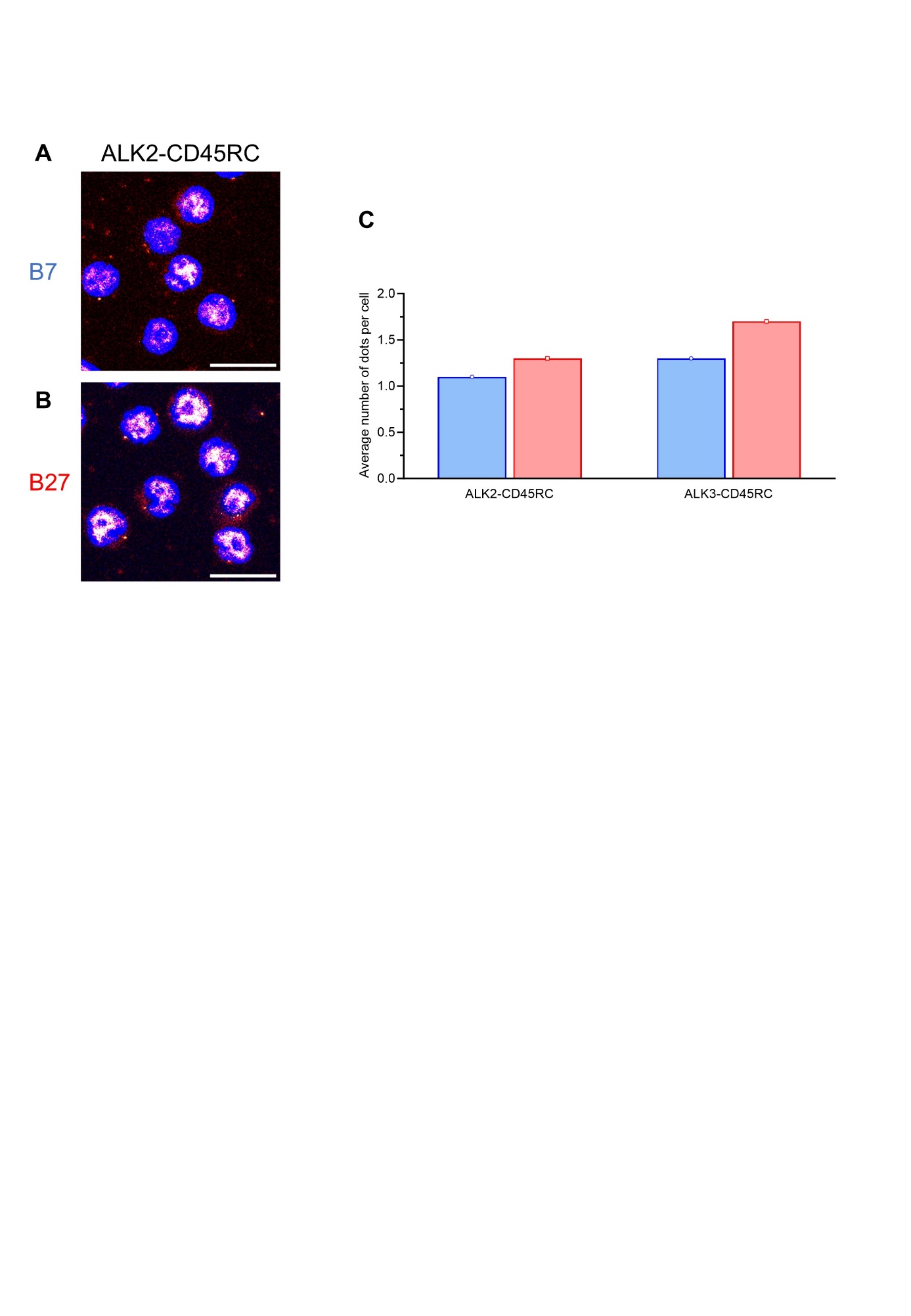


**Supplementary Fig. 2**: **TGFβ, not BMP nor activin, induced pSMAD, with heightened change in B27 rat T cells.** (A) Intracellular pSMAD1/5/8 was quantified in CD3+ T cells from B7 rat after exposure to BMP2/6 mixture (200ng/mL each) for 1h to 24h (left panel) or various concentrations of BMP2 or BMP6 (250 to 1000ng/mL) for 1h (right panel). (B) Intracellular pSMAD2/3 was quantified in (from left to right) bulk CD3+ T cells, sorted Tn cells and sorted Teff cells from NTG or B27 adult rats after 1h treatment with activin. Scattered dot plots with bars show the fold change mean out of 5 to 7 experiments calculated as the ratio between stimulated (activin) and unstimulated (PBS) conditions. Similar symbols indicate paired conditions. Vertical error bars show SEM. The horizontal dashed line corresponds to a ratio of 1. Ratio above this line means that SMAD2/3 phosphorylation was induced by activin treatment. (C) Gating strategy in experiments using bulk CD3+ T cells. (D) Intracellular pSMAD2/3 was quantified after exposure to PBS or TGFβ in sorted Teff from B27 and NTG adult rats. Scatter dot plots graphic with bars show SI mean out of 8 independent experiments. Similar symbols indicate paired conditions. Vertical error bars show the SEM. (E) Gating strategy to analyse Tn and Teff. (F) Fold change of SMAD2/3 phosphorylation after exposure to TGFβ1 in bulk CD3+ mLN T cells from adult rats. Scattered dot plots with bars show the mean of the fold change out of 8 independent experiments. Similar symbols indicate paired conditions. (G) Fold change of SMAD2/3 phosphorylation after exposure to TGFβ1 in gated Tn and Teff from premorbid rats. Scattered dot plots with bars show the mean of the fold change out of 7 independent experiments. Similar symbols indicate paired conditions. Vertical error bars show the SEM. Paired t-test were performed. *: p<0.05; **: p<0.01.


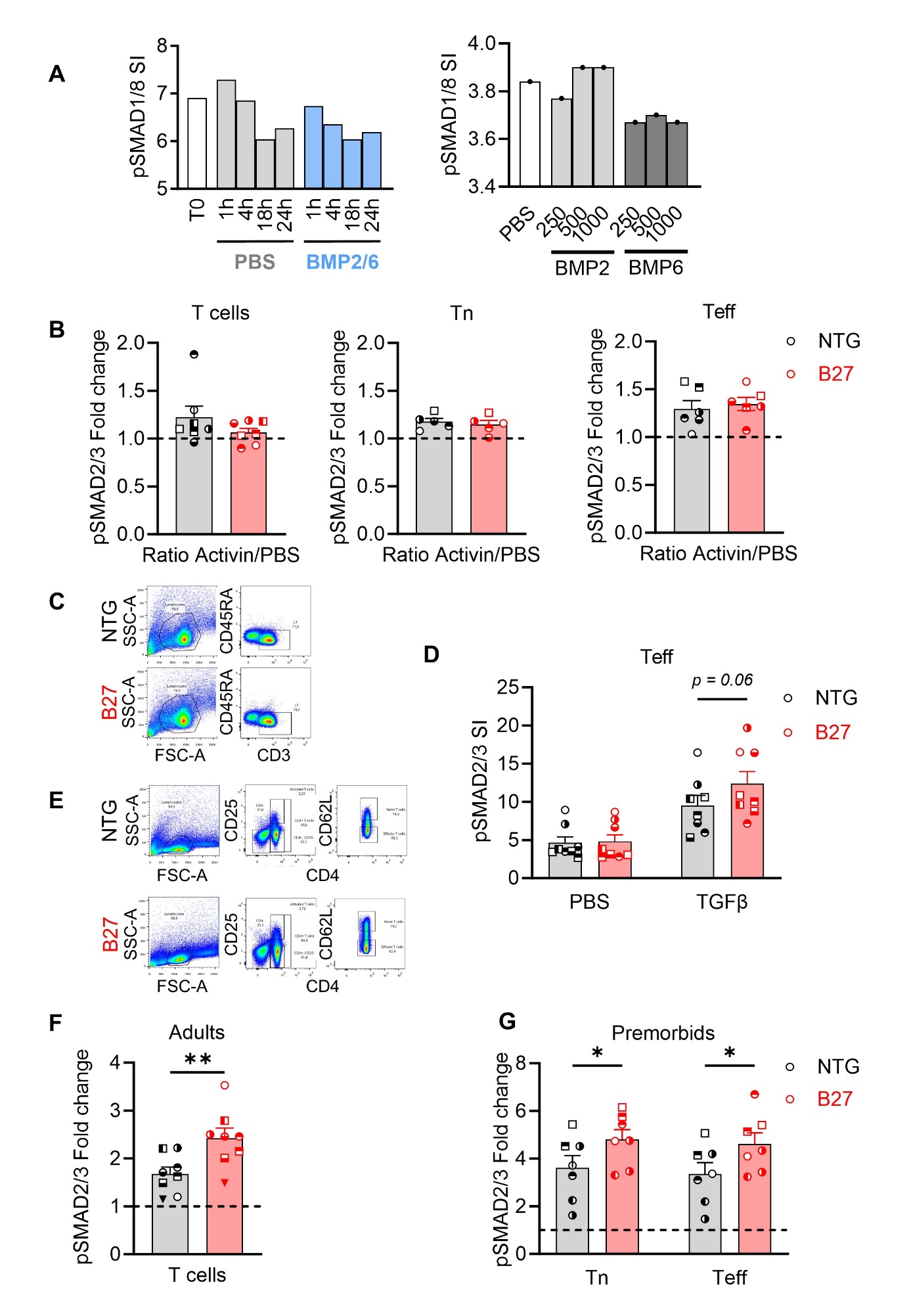


**Supplementary Fig. 3**: **TGFβ1 fails to induce phosphorylation of non-canonical pathways in rat CD4+ T cell subsets.** (A) Intracellular p-p38, p-AKT or p-ERK was assessed after 5 or 30 min of TGFβ1 exposure in sorted Tn from adult NTG and B27 rats. Scattered dot plots with bars show the mean of the fold change from 2 to 3 independent experiments. The red dashed line indicates a ratio = 1 showing that the treatment had no effect on phosphorylation levels. (B) *Ex-vivo* intracellular staining of p-p38, p-AKT, p-ERK, p-mTOR and p-NF-kB was evaluated in CD4+ T cells subpopulations (Tn and Teff) from premorbid NTG and B27 rats. Scattered dot plots with bars show the mean of the SI from 5 to 11 independent experiments. 2-way ANOVA with Geisser-Greenhouse correction (factors: cell type, rat strain) followed by Fischer’s multiple comparison was performed. *: p<0.05; **: p<0.01.


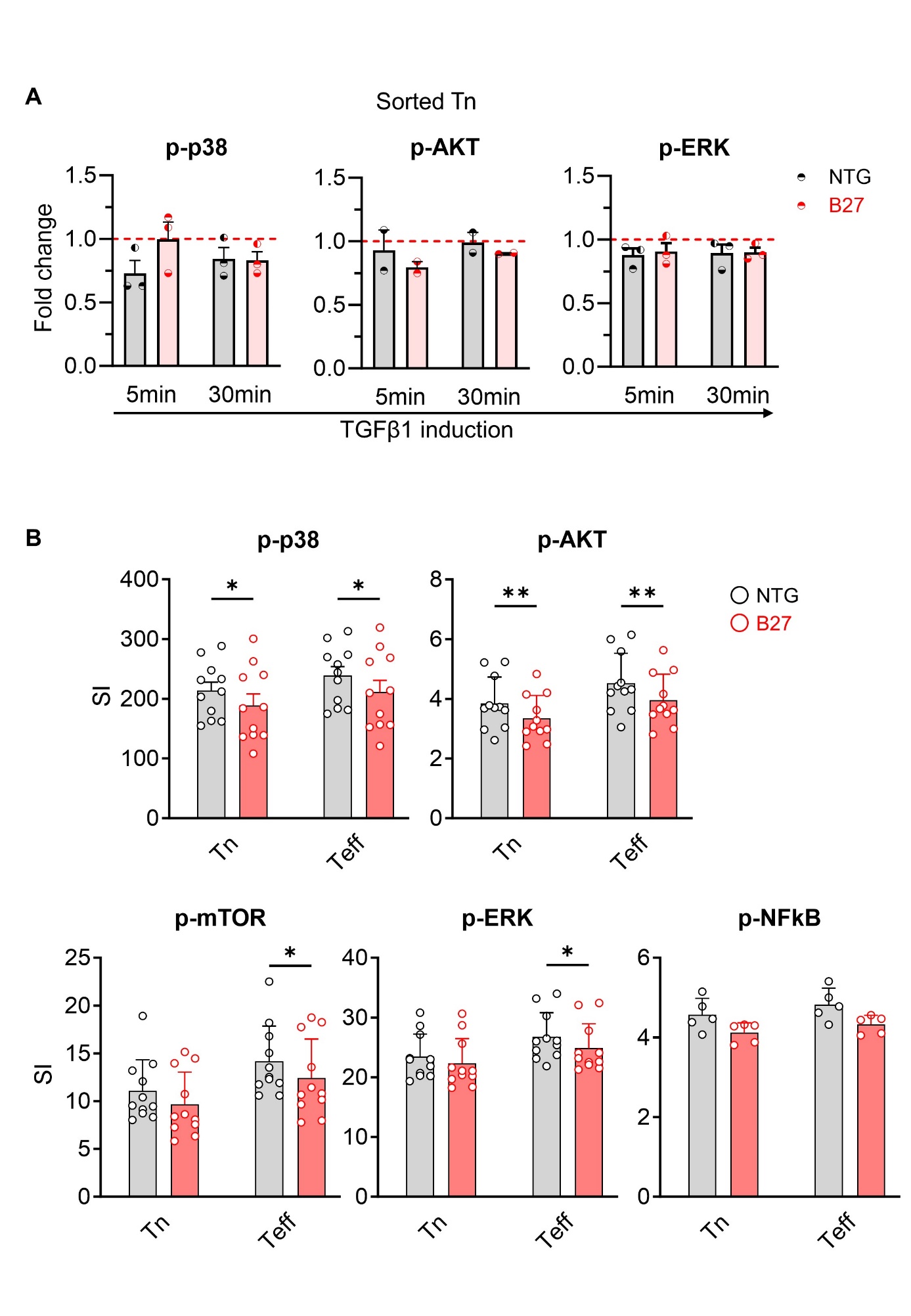


**Supplementary Table 1: *Drosophila* genotypes**

| Genotype | Provenance |
| --- | --- |
| *w^1118^  : control strain* | Provided by M. Hoareau |
| *nub-Gal4 ; UAS-eGFP* | Provided by M. Hoareau |
| *nub-Gal4 ; UAS HLAB2705 (2M), β2m (3M)* | This study |
| *UAS-LUC* | BDSC BL-35788 |
| *UAS-Actβ RNAi* | BDSC BL-29597 |
| *UAS-babo RNAi* | BDSC BL-25933 |
| *UAS-Medea RNAi* | BDSC BL-31928 |
| *UAS-Smox RNAi* | BDSC BL-26756 |

The yeast GAL4 transcription factor is placed under the control of *Drosophila* nubbin (nub) gene promotor, allowing the transcriptional activation of different transgenes placed under the control of upstream activating sequence (UAS), specifically in the nub gene expression domain (i.e. the larval imaginal wing disc pouch, a structure that gives rise to the adult wing mainly)

LUC : luciferase. Luciferase and GFP are used as controls and have no effects on wing formation

Actβ : activin beta ligand of the activin/TGF beta pathway

Babo : Baboon type I receptor of the activin/TGF beta pathway, the *Drosophila* ortholog of ALK5

Medea : co-Smad involved in both BMP and activin/TGF beta pathways, the *Drosophil*a ortholog of SMAD4

Smox : Smad on X, the Drosophila ortholog of SMAD2

BDSC : Bloomington Drosophila Stock Center

**Supplementary Table 2: Reagents and Abs**

| **Medium** | **Catalog No.** | **Provider** |
| --- | --- | --- |
| **Recombinant cytokines** |  |  |
| TGF-β1 (rh) | 130-095-066 | Miltenyi Biotec |
| Activin A (h/r/m) | 338-AC-010 | Biotechne |
| BMP-4 (rh) | 130-111-164 | Miltenyi Biotec |
| BMP-6 (rh) | 130-112-951 | Miltenyi Biotec |
| BMP-2 (rh) | PHC7145 | Thermo Fisher |
| IL-2 (rr) | 502-RL-010 | Miltenyi Biotec |
| IL-23 (rr) | 3136-RL | R&D systems |
| IL-1 (rr) | 501-RL-010 | R&D systems |
| IL-6 (rr) | 506-RL-010 | R&D systems |
| **Purified Abs** |  |  |
| Anti-ACVRL1 (ALK1) | AB4531 | Antibodies |
| Anti-ACVR1 (ALK2) | SAB2108587 | Sigma-Aldrich |
| Anti-BMPR1A (ALK3) | 38-6000 | Invitrogen |
| Anti-TGFBRI (ALK5) | SAB5700651 | Sigma-Aldrich |
| Anti MHC-I (w6.32) | ab22432 | Abcam |
| Anti-IL-4 | 16-704582 | ThermoFisher |
| Anti-IFN gamma | 14-7310-85 | ThermoFisher |
|  |  |  |
| **Conjugated Abs** |  |  |
| Anti-Smad2/3 PE | 72255 | Cell Signaling |
| Anti-rat CD4 V450 | 561579 | BD Biosciences |
| Anti-rat CD62L APC | 50-0623-82 | Thermo Fisher |
| Anti-rat CD25 PE | 12-0390-82 | Thermo Fisher |
| Anti-rat CD25 FITC | MA5-17573 | Thermo Fisher |
| Anti-rat CD4 FITC | 11-0040-85 | Thermo Fisher |
|  |  |  |
| **Phospho conjugated Abs** |  |  |
| Anti-Smad2 (pS465/pS425) and Smad3 (pS423/pS425) PE | 562586 | BD Biosciences |
| Anti-Smad1 pS463/pS465 and Smad8 (pS465/pS467) PE | 562509 | BD Biosciences |
| Anti-AKT (pS473) AF488 | 4071 | Cell Signaling |
| Anti-ERK1/2 (pT202/pT204) Pe-eF610 | 61-9109-42 | Invitrogen |
| Anti p38 MAPK (pT180/pT182) PE-Cy7 | 25-9078-42 | Invitrogen |
| Anti-phospho NF-κB p65 (pS536) PE | 5733 | Cell Signaling |
|  |  |  |
| **Other reagents** |  |  |
| FICOLL 400 | F4375 | Sigma-Aldrich |
| Fix Buffer I | 557870 | BD Biosciences |
| Fix Buffer III |  |  |
| Perm Wash 10X | 554723 | BD Biosciences |
| Foxp3 / Transcription Factor Fix/Perm Diluent (1X) | TNB-1022-L160 | Thermo Fisher |
| Foxp3 / Transcription Factor Fix/Perm Concentrate (4X) | TNB-1020-L050 | Thermo Fisher |
|  |  |  |
| **PLA reagents** |  |  |
| Anti-rabbit probe PLUS | DUO92002 | MERCK |
| Anti-mouse probe MINUS | DUO92004 | MERCK |
| Detection regeants | DUO92008 | MERCK |
| ProLong Diamond antifade mountants with DAPI | P36971 | Thermo Fisher |
|  |  |  |
| **RT-qPCR** |  |  |
| Reverse Aid | K1622 | Thermo Fischer |
| TRIzol | 12034977 | Thermo Fischer |
| SsoAdvanced™ Universal SYBR® Green Supermix | 1725274 | Bio-rad |

**Supplementary Table 3 : Primers sequence**

| ***Gene*** | **Forward primer** | **Reverse primer** |
| --- | --- | --- |
| *Gapdh* | AGCCCAGCAAGGATACTGAG | GGATGGAATTGTGAGGGAGA |
| *Acvr1* | GAACGGACAGTGCTGCATAG | AAGGCCCAAATATCGACCCT |
| *Bmpr1a* | CCAGACGGTGTTAATGCGTC | CCACAGGCAGCAGAATAAGC |
| *Acvr1b* | CTGCGCCATGAAAACATCCT | CAATGGTCACTGTGTAGCGG |
| *Tgfbr1* | GAGAAGTTTGGCGAGGCAAA | CAACCACAGCTGAGTCCATG |
| *Acvr1c* | TGTGGAGAAGATGTGGCTGT | AAAGCGCCAACTTGACCATT |
| *Acvr2a* | TCAGACTGGTGTTGAGCCTT | GTGTGACTTCCATCTCCGGA |
| *Acvr2b* | AGTTGCTGGAGATCAAGGCT | TGAAGATCTCCCGCTCACTC |
| *Bmpr2* | GGTGGCACGTGTGTTATCAG | TCACAGTCCCTCAGGTTCAC |
| *Runx1* | CCCTCAGCCTCAAAGTCAGA | GAAAGTTCCGCTGAGAGGCT |
| *Foxp3* | CTCTGGGGAAGCCATGGAAA | ATCGGATGAGGGTGGCATAG |
| *Rorc* | TACACGGCCTTGGTTCTCAT | GCAGATGCTCCACTCTCCTC |
| *Maf* | GAGAAGTTGGTGAGCAGCG | TCACAGATGTAGAAGAGTCCCT |
| *Smad7* | TCCAGACGCTGTACCTTCCT | CAGGCTCCAGAAGAAGTTGG |
| *Tgfb1* | GACTCTCCACCTGCAAGACC | CGAGCCTTAGTTTGGACAGG |
